# Melatonin regulates autophagy in granulosa cells from patients with premature ovarian insufficiency via activating Foxo3a

**DOI:** 10.18632/aging.205424

**Published:** 2024-01-10

**Authors:** Pengfen Li, Qian Dou, Dan Zhang, Yungai Xiang, Li Tan

**Affiliations:** 1 Department of Reproductive Center of The Second Affiliated Hospital of Zhengzhou University, Zhengzhou 450014, Henan Province, China

**Keywords:** melatonin, autophagy, granulosa cells, premature ovarian insufficiency, forkhead box O-3A

## Abstract

Premature ovarian insufficiency (POI) is a diverse form of female infertility characterized by a decline in ovarian function before the age of 40. Melatonin (MT) is a potential clinical treatment for restoring or safeguarding ovarian function in POI. However, the specific therapeutic mechanism underlying this effect remains unclear. To address this, we conducted experiments using human granulosa cells (GCs) from both POI and normal patients. We examined the expression levels of autophagy-related genes and proteins in GCs through qRT-PCR and western blot analysis. Autophagy flux was monitored in GCs infected with GFP-LC3-adenovirus, and the regulatory function of MT in autophagy was investigated. Additionally, we employed pharmacological intervention of autophagy using 3-Methyladenine (3-MA) and RNA interference of Forkhead box O-3A (FOXO3A) to elucidate the mechanism of MT in the autophagy process. Compared to GCs from normal patients, GCs from POI patients exhibited irregular morphology, decreased proliferation, increased apoptosis, and elevated ROS levels. The expression of autophagy-related genes was downregulated in POI GCs, resulting in reduced autophagic activity. Furthermore, MT levels were decreased in POI GCs, but exogenous MT effectively activated autophagy. Mechanistically, melatonin treatment downregulated FOXO3A expression and induced phosphorylation in POI GCs. Importantly, silencing FOXO3A abolished the protective effect of melatonin on GCs. These findings indicate that autophagy is downregulated in POI GCs, accompanied by a deficiency in MT. Moreover, we demonstrated that supplementing MT can rescue autophagy levels and enhance GC viability through the activation of FOXO3A signaling. Thus, MT-FOXO3A may serve as a potential therapeutic target for POI treatment.

## INTRODUCTION

Premature ovarian insufficiency (POI) is a type of female infertility characterized by the premature decline of ovarian function, resulting in elevated gonadotropins and decreased estradiol concentrations before the age of 40 years [1, 2]. It significantly impacts women's physical and mental health. Moreover, the incidence of POI has been steadily increasing, particularly among younger women. While the overall incidence of POI in women is around 1%, it rises to 10%-28% in women with primary amenorrhea [3]. However, POI is a highly heterogeneous disease with various causes, including genetic, immune, iatrogenic, infection, environmental, and personal factors. Consequently, the etiology and pathogenesis of the majority of POI cases remain unclear [1–3].

GCs are one of the functional cells of follicle, secreting sex hormones, growth factors and cytokines to regulate the growth and development of follicles [4, 5]. The disorder and dysfunction in GCs’ biological process will affect the normal follicular development, maturation and ultimately affect fertility. Previous studies have demonstrated that the apoptosis of GCs is the main cause of follicular atresia [6–8]. Therefore, investigating the role and mechanism of GCs in the pathogenesis of POI will offer a novel perspective and valuable insights into the etiology of POI.

Besides of apoptosis, autophagy is another programmed self-digestion process in cells which is closely participated in a variety of processes such as development, metabolism, immune regulation and aging to keep cell homeostasis and balance [9–13]. Previous studies have shown that autophagy is also involved in the establishment and maintenance of follicle reserve, the physiological process of ovarian oocyte aging, and the pathological process of Premature Ovarian Failure (POF), POI and polycystic ovary syndrome (PCOS) [14–16]. When the level of autophagy of GCs accumulates, the programmed apoptosis program is activated, which causes GC apoptosis and eventually causes follicular atresia, which promotes the occurrence of POI [17–20]. Therefore, studying the role and mechanism of GC autophagy in the pathogenesis of POI will provide a new target for the early identification and intervention of POI.

N-acetyl-5-methoxytryptamine (Melatonin, MT) is an indoleamine hormone, which can be directly synthesized by the human ovary and released into the follicular fluid to regulate oxidative stress via inhibiting apoptosis and autophagy thereby protecting oocytes and GCs [21–23]. Dietary administration of MT delays ovarian aging in mice by improving the number of follicles, increasing the fertilization rate of oocytes and the rate of blastocyst formation [24–26]. More important, following studies have found that MT treatment shown a protective effect on POI [27–30] and some key targets which downstream of MT were elucidated, such as phosphatidylinositol-3-kinase (PI3K)-Akt-mammalian target of rapamycin (mTOR) [31], YAP-Hippo [32], silent information regulator 1 (SIRT1) pathway [33] and so on. Therefore, gaining a comprehensive understanding of the mechanisms by which MT regulates GC autophagy is crucial in developing effective clinical treatments aimed at restoring or protecting ovarian function.

In this study, we isolated GCs from both POI and normal patients, and observed that the POI-GCs exhibited irregular morphology, reduced proliferation, and increased apoptosis ratio with elevated ROS levels. Moreover, we found that the expression of genes and proteins related to autophagy were impaired in the POI group compared to the normal group, while MT levels were significantly lower in the former, indicating MT deficiency as a potential cause for down-regulated autophagy. To investigate this further, we treated POI-GCs with exogenous MT at varying concentrations and observed that it effectively reversed the levels of autophagy-related proteins and improved cell viability. Additionally, we found that Forkhead box O-3A (FOXO3A), a downstream molecule of MT known to modulate autophagy, was down-regulated in the POI group. However, upon treatment with MT, we observed a significant concentration gradient-dependent activation of FOXO3A protein levels, which were abolished upon silencing FOXO3A, suggesting the MT-FOXO3A pathway as a potentially promising genetic target for POI therapy.

## MATERIALS AND METHODS

### Patients

The study population consisted of patients who underwent *in vitro* fertilization (IVF) treatment at the Reproductive Medicine Center, The Second Affiliated Hospital of Zhengzhou University, from January 2022 to May 2023. Inclusion criteria for the POI patients: age < 35 years and meeting the diagnostic criteria for POI, which include being < 40 years of age, experiencing oligomenorrhea or amenorrhea for at least 4 months, and having a basal serum follicle-stimulating hormone (FSH) level ≥ 25 IU/L measured at least 2 times with an interval of > 4 weeks [34]. Patients with normal ovarian reserve who underwent IVF treatment due to tubal or male factor infertility were included as the normal group [34]. The inclusion criteria for normal patients were < 35 years of age, regularly previous menstruation, no history of hormone therapy in the past 3 months, basal serum FSH from 5 to 10 IU/L, AMH > 1.0 ng/ml, no PCOS-relative diseases and excluding patients with ovarian hyperstimulation syndrome, HCG daily estrogen < 4000pg/mL, ovum ≤ 15. Any women who was BMI > 25kg/m^2^ or < 18.5 kg/m^2^, diagnosed with chromosomal abnormalities, amenorrhea caused by abnormal genital development or other diseases (such as tumor, obesity, thyroid dysfunction, adrenal dysfunction and so on), uterine amenorrhea and physiological amenorrhea, suffering from malignant tumors, mental disorders, endometriosis, autoimmune disorders, with a history of ovarian surgery, radiotherapy, or chemotherapy/previously recurrent implantation failure/recurrent miscarriage, or taking antioxidant agents in the past three months were excluded. The study protocol was approved by the ethics committee of the Second Affiliated Hospital of Zhengzhou University. All patients signed written informed consent.

### Human GC isolation and culture

Transvaginal ultrasound-guided follicular aspiration was performed to retrieve the follicular fluid after the cumulus-oocyte complex was identified and selected in the embryology laboratory, the follicular fluid containing mural GCs was collected and preserved in 4° C until use. The precipitates of follicular fluid were separated by centrifugal gradient method at 3000rpm for 10min in 4° C. Discard the supernatant, carefully absorb the sticky white (or pink) part on the top layer of the precipitate into a new centrifuge tube, add appropriate phosphate buffered saline (PBS), blow and mix well, and centrifuge at 3000rpm for 8min in 4° C. The supernatant was discarded, and appropriate PBS was added, blown and mixed. The mixture liquid was spread on the surface of human blood lymphocyte separation solution according to the volume ratio of 1:2, and centrifuged at 2000rpm for 30min in 4° C. After centrifugation, the liquid was divided into four layers from top to bottom: colorless transparent PBS layer, granule cell layer (white cloudy cell suspension), light yellow lymphocyte separation solution layer, and red blood cell layer. The cell suspension of granule cell layer was carefully aspirated, placed in a centrifuge tube with appropriate PBS, mixed and centrifuged at 3000rpm for 10min in 4° C. Finally, part of the cell precipitate was placed in an EP tube and frozen in an ultra-low temperature refrigerator. Part of the precipitate was added into the cell culture medium, and the granule cells were cultured in 37° C, 5% CO_2_ incubator.

### Flow cytometry (FACS) analysis of cell apoptosis

FACS for detecting cell death was performed as described previously [23]. Harvested cells were stained with Annexin V-FITC and PI (Beytime, C1062) then analyzed by flow cytometer. Flowjo7.6 software was utilized to analyze the FACS data.

### Detection of reactive oxygen species (ROS) production

ROS levels were determined using the Reactive Oxygen Species Assay Kit (Beyotime, S0033) according to the manufacturer’s instructions. The GCs were imaged with confocal microscope and fluorescence intensity was calculated by using the Image Processing and Analysis software in Java (Image J).

### GFP-LC3 adenovirus preparation and adenoviral transduction

The autophagic flux was detected as previously reported [35, 36]. In brief, the infection of GCs with the tandem GFP-LC3 reporter adenovirus (Beyotime, C3006) was performed according to the manufacturer's protocol. For investigation the function of MT in regulating autophagy, different doses of MT were added into POI GC-medium after 48h of adenovirus infection. Then GCs were harvested and cell nuclei were stained with Hoechst 33342 (Beyotime, C1029) for 10 min.

### EdU assay

EdU Cell Proliferation Kit (Beyotime, C0071) was used to detected GC proliferation according to the manufacturer’s instructions. The images were blindly captured by using a Zeiss inverted microscope and three microscopic fields of each slide were quantified for each group with Image J.

### RNA extraction and quantitative reverse transcription polymerase chain reaction (qRT-PCR)

Total RNA of all samples was extracted with Trizol reagent (Thermo Fisher Scientific, 15596026) via phenol-chloroform precipitation. After treatment with DNase I (Promega) to eliminate genomic DNA, total RNA was then reverse transcribed to cDNA using oligo (dT) primer and reverse transcriptase (Takara, RR047A). The qRT-PCR was performed by using ABI PRISM 7900 system (Applied Biosystems) with the SYBR Green Real time PCR Master Mix plus (TOYOBO). GAPDH was used as the internal normalization. The primers used for qRT-PCR are listed below: human GAPDH (Forward: 5’- CACCCAGAAGACTGTGGATGG -3’, Reverse: 5’- GTCTACATGGCAACTGTGAGG -3’); human FOXO3A (Forward: 5’- GCGTGCCCTACTTCAAGGATAAG -3’, Reverse: 5’- GACCCGCATGAATCGACTATG -3’); human Autophagy Related 7 (ATG7) (Forward: 5’- CTGCCAGCTCGCTTAACATTG -3’, Reverse: 5’- CTTGTTGAGGAGTACAGGGTTTT -3’); human Beclin 1 (BECN1) (Forward: 5’- CCATGCAGGTGAGCTTCGT -3’, Reverse: 5’- GAATCTGCGAGAGACACCATC -3’); and human Microtubule-Associated Protein 1 Light Chain 3 Beta (LC3B) (Forward: 5’- CGGAGAAGACCTTCAAGCAG-3’, Reverse: 5’- ctgggaggcatagaccatgt -3’).

### Western blot

Total protein from all samples was harvested with RIPA lysis buffer (Beyotime, P0013B), the protein concentration was determined using a BCA kit (Beyotime, P0010). The protein components were separated using SDS-PAGE and transferred to polyvinylidene fluoride membranes (Bio-Rad, USA). After blocking with 5% skim milk, the membranes were incubated with primary antibodies overnight in 4° C and then incubated with appropriate secondary antibody conjugated with horseradish peroxidase for 1 h at room temperature. The antibody information used in this study is as follows: Anti-Beclin 1 (1:2000, CST, #3495), Anti-LC3II (1:1000, Proteintech, 14600-1-AP), Anti-ATG7 (1:2000, CST, #8558), Anti-SQSTM1/P62 (1:2000, Proteintech, 18420-1-AP), Anti-FOXO3A (1:2000, CST, #12829), and Anti-GAPDH (1:8000, Proteintech, 60004-1-Ig). The detection and analysis of the protein bands were visualized following activation with ECL (Thermo Fisher Scientific, USA) and exposure on film (Kodak Carestream Biomax, Sigma-Aldrich, USA).

### Enzyme-linked immunosorbent assay (ELISA)

GCs from POI and normal patients were used to test MT expression levels with ELISA kits (Sangon Biotech, Shanghai, China) and all procedures were conducted according to the manufacturer’s instructions.

### Statistical analysis

The statistical analyses were performed with GraphPad Prism software (version 8.0). All data are presented as mean ± SD. Comparisons of means between two groups were analyzed by using unpaired Student’s t-test, while comparisons between more than two groups were made by one-way ANOVA followed by t-test. The differences were considered to be significantly different when the P-value was less than 0.05.

### Data availability statement

The data that support the findings of this study are available from the corresponding author upon reasonable request.

## RESULTS

### Autophagy is attenuating in GCs from POI patients

**Figure 1:**
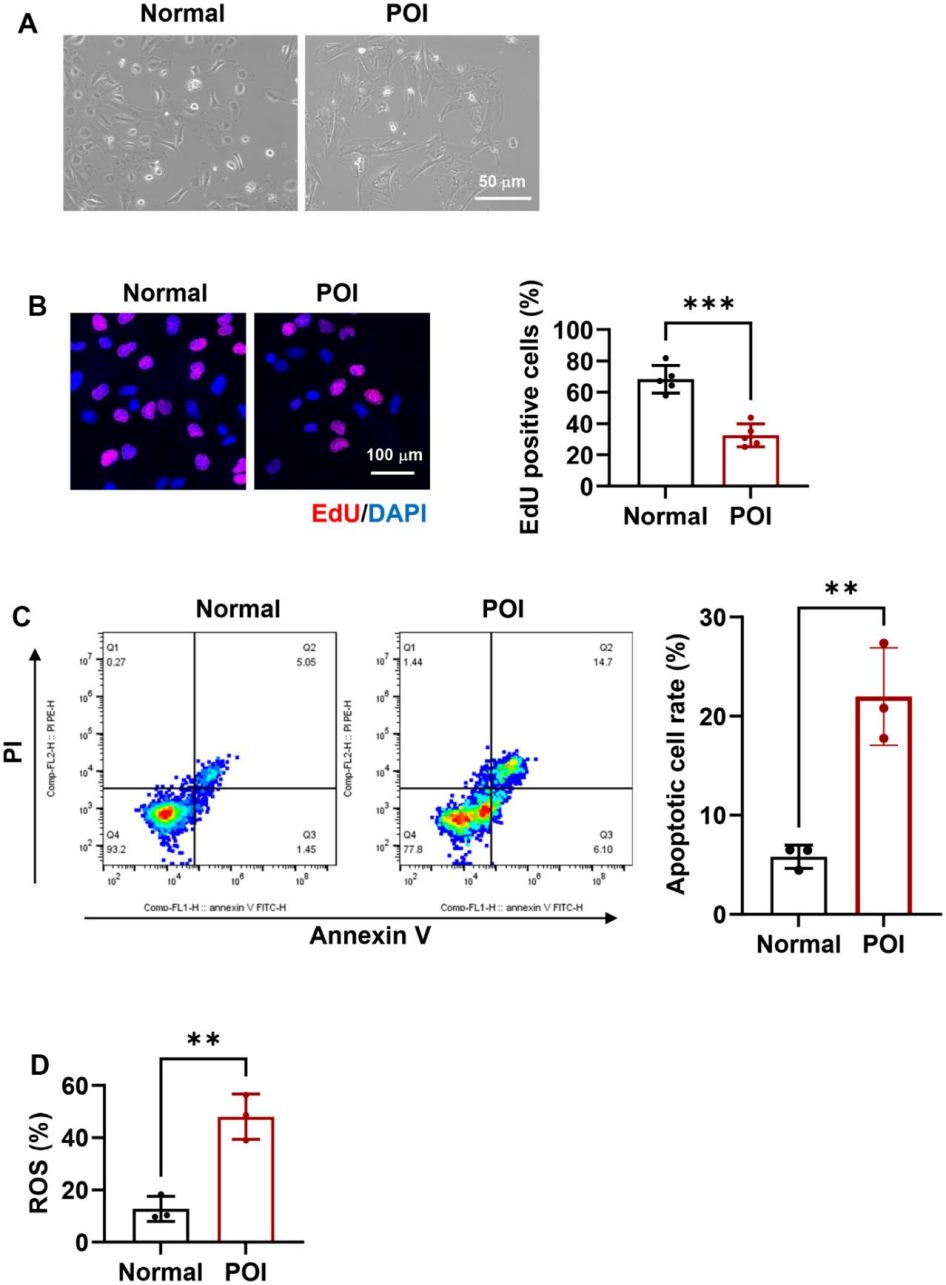
**Morphological and functional characteristics of granulosa cells (GCs) in premature ovarian insufficiency (POI) patients and normal controls.** (**A**) Microscopic images depicting the morphology of GCs. Scale bar, 50 μm. (**B**) Representative immunofluorescence (IF) staining and analysis of cell proliferation in GCs (n = 5). Scale bar, 100 μm. (**C**) Flow cytometry analysis of apoptotic rates in GCs (n = 3). (**D**) Analysis of reactive oxygen species (ROS) production in GCs. **p < 0.01, ***p < 0.001.

**Figure 2:**
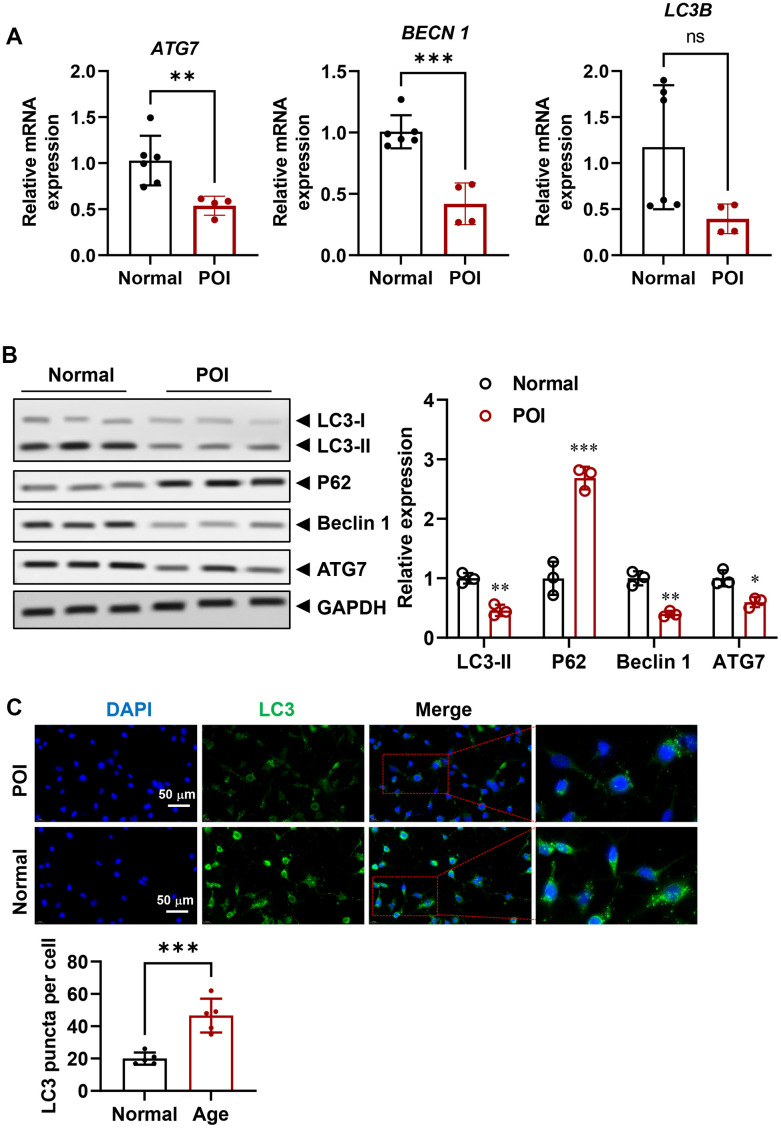
**Attenuated autophagy in GCs from POI patients.** (**A**) mRNA levels of autophagy-related genes in GCs (n = 4-6). (**B**) Representative Western blots (WB) and analysis of autophagy-related proteins in GCs (n = 3). (**C**) Representative IF staining and analysis of autophagy flux in GCs (n = 5). Scale bar, 50 μm. ns, no significant, *p < 0.05, **p < 0.01, ***p < 0.001.

Compared to GCs from normal patients, POI-GCs exhibit irregular morphology (Figure 1A), suggesting a change in cellular status. To investigate further differences in cell viability, we examined cell proliferation and survival. POI-GCs exhibited a lower ratio of EDU-positive cells but a higher apoptotic rate compared to the normal group (Figure 1B, 1C). These findings were supported by an elevation in cellular ROS levels (Figure 1D). Previous reports have indicated the significance of autophagy in the process of POI [2, 16, 18, 36]. Therefore, we initially examined the variation in autophagy levels between POI and normal GCs. We observed a down-regulated tendency in genes related to autophagy, such as ATG7, BECN1, and LC3B, in the POI group compared to the normal group. Notably, ATG7 and BECN1 displayed significant differences (Figure 2A). Consistent with qRT-PCR results, the protein levels of these autophagy-related genes were significantly decreased (Figure 2B). Furthermore, the accumulation of SQSTM1/p62 also indicated a down-regulation of autophagy in POI-GCs (Figure 2B). To evaluate the autophagic flux, we infected GCs with a GFP-LC3 reporter adenovirus, where the GFP signal is quenched in autolysosomes. Immunofluorescence results demonstrated a significant reduction in the green-LC3 signal in POI-GCs compared to the normal group, indicating attenuated autophagic flux (Figure 2C).

### MT is down-regulating in POI-GCs and MT treatment improves cell viability via activating autophagy

**Figure 3:**
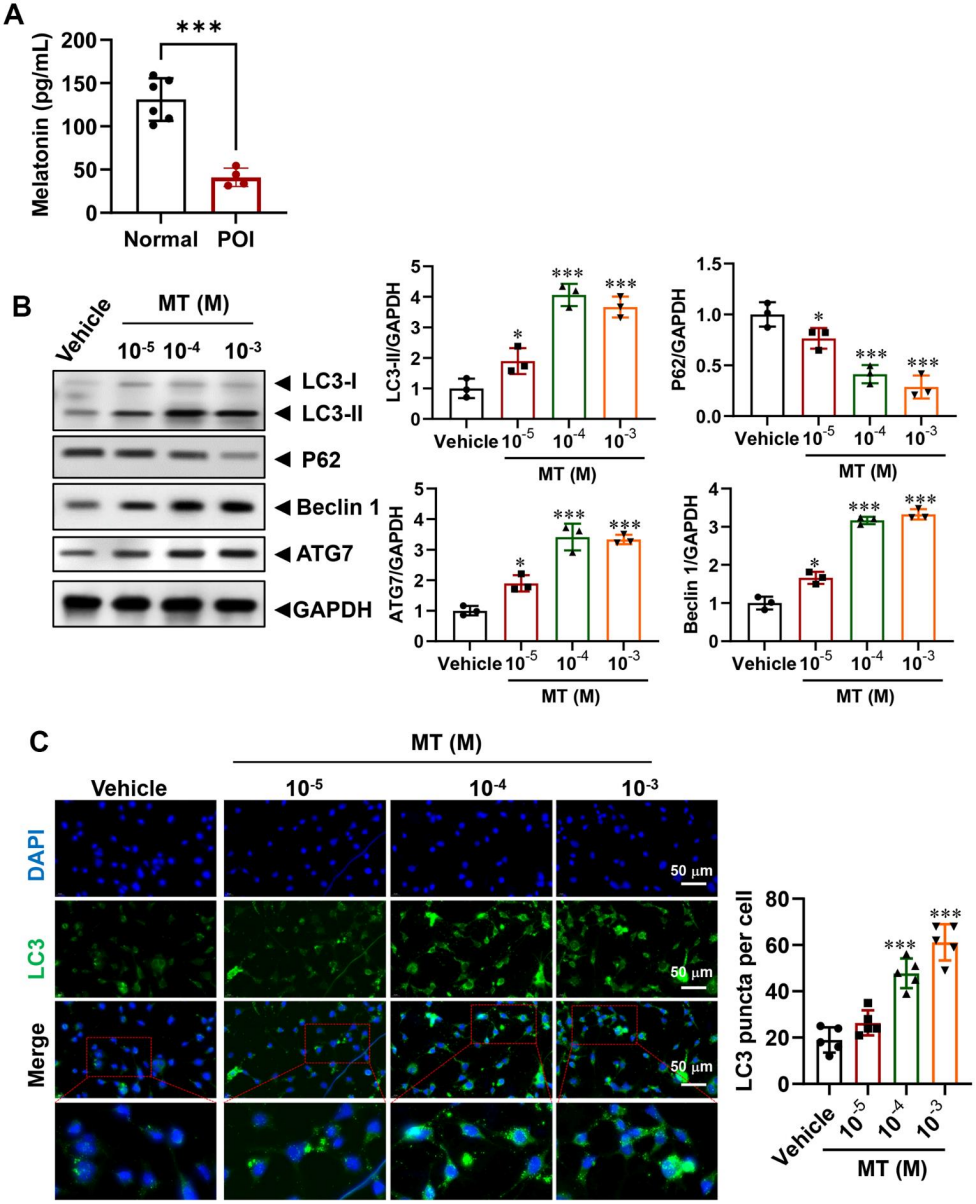
**Regulation of autophagy in POI-GCs by melatonin (MT).** (**A**) ELISA analysis of MT levels in GCs (n = 4-6). (**B**) Representative WB and analysis of autophagy-related proteins in POI-GCs after MT treatment (n = 3). (**C**) Representative IF staining and analysis of autophagy flux in POI-GCs after MT treatment (n = 5). Scale bar, 50 μm. *p < 0.05, **p < 0.01, ***p < 0.001.

Although the role of MT in regulating autophagy has been partially elucidated [23, 26, 31], its expression level in POI-GCs remains unknown. To investigate this, we measured MT levels via ELISA and found that they were significantly decreased in the POI group compared to the non-POCS group (Figure 3A). To establish the relationship between MT deficiency and down-regulated autophagy in POI, we treated POI-GCs with exogenous MT and found that MT efficiently reversed the levels of autophagy-related proteins in a concentration-dependent manner (Figure 2B). Additionally, IF staining analysis demonstrated that autophagic flux was activated in POI-GCs after MT treatment (Figure 3C).

**Figure 4:**
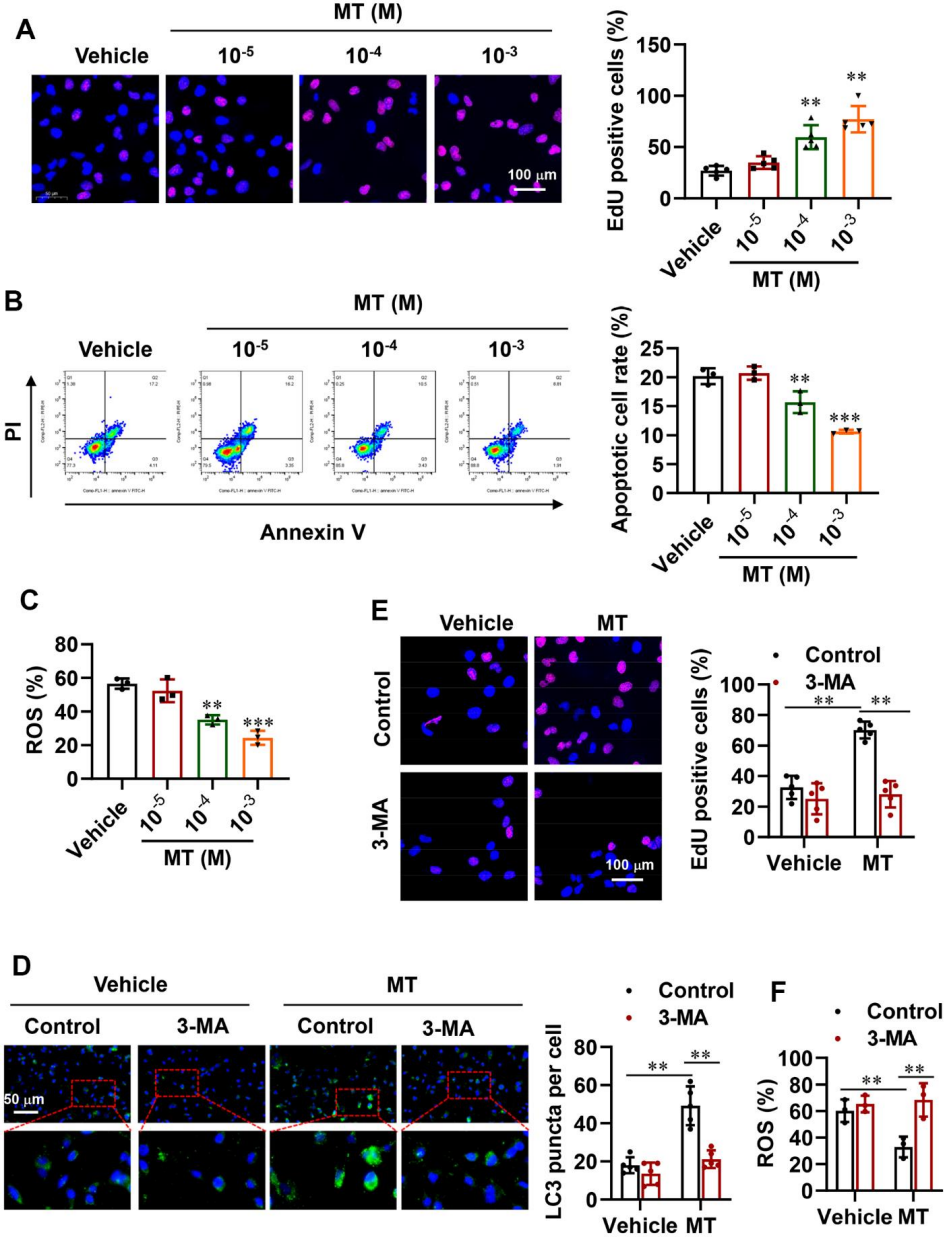
**Improvement in cell proliferation and survival via activated autophagy with MT treatment.** (**A**) Representative EdU staining results and analysis for cell proliferation in POI-GCs after MT treatment (n = 5). Scale bar, 100 μm. (**B**) Flow cytometry analysis of apoptotic rates in POI-GCs after MT treatment (n = 3). (**C**) Analysis of ROS production in POI-GCs after MT (n = 3). (**D**) Representative IF staining and analysis of autophagy in POI-GCs after 3-MA treatment with or without MT (n = 5). (**E**) EdU results of POI-GCs. (**F**) Analysis of ROS production in POI-GCs after 3-MA treatment with or without MT. **p < 0.01, ***p < 0.001.

To determine whether activating autophagy in POI-GCs via MT treatment is beneficial, we assessed cell proliferation and survival in these cells. Encouragingly, we observed an increase in cell proliferation rate (Figure 4A), while cell apoptosis and ROS levels were dramatically decreased after MT treatment (Figure 4B, 4C). We also disrupted autophagy in GCs pharmacologically. Specifically, we used 3-Methyladenine (3-MA), an autophagy inhibitor that selectively targets class III phosphatidylinositol 3-kinase (PtdIns3K), which has been reported to affect glucose metabolism and induce apoptosis in GCs. The fluorescence results showed that green LC3 puncta were significantly increased with MT treatment, while this effect was reversed by 3-MA (Figure 4D). Consistently, 3-MA abolished the promotion of GC proliferation by MT and further promoted cellular oxidative stress levels (Figure 4E, 4F).

### MT reverses autophagy insufficiency in POI-GCs via FOXO3A signaling

**Figure 5:**
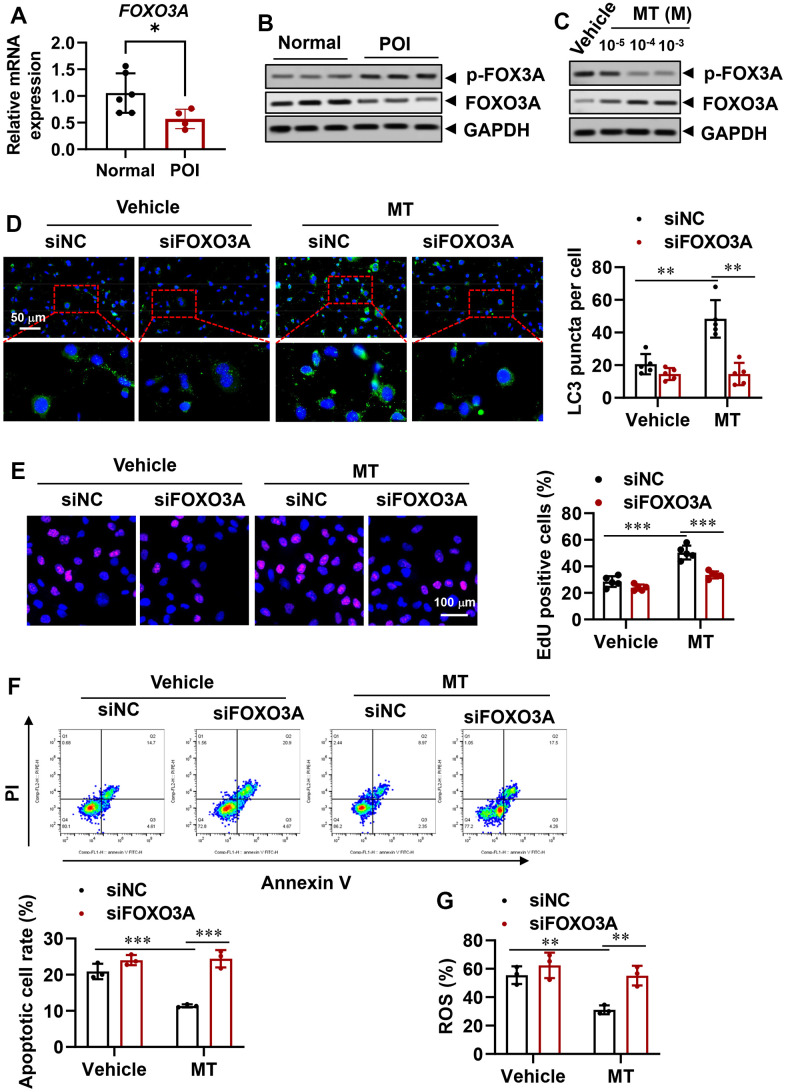
**Reversal of autophagy insufficiency in POI-GCs by melatonin via forkhead box O-3A (FOXO3A) signaling.** (**A**) Gene expression of FOXO3A in GCs from POI and normal patients (n = 4-6). (**B**) Representative WB results of FOXO3A in GCs from POI and normal patients (n = 3). (**C**) Representative WB of FOXO3A in POI-GCs after MT treatment (n = 3). (**D**) Representative IF staining and analysis of autophagy in POI-GCs after FOXO3A knockdown with or without MT (n = 5). (**E**) Representative EDU staining results and analysis for cell proliferation in POI-GCs after MT and siFOXO3A treatment (n = 5). Scale bar, 100 μm. (**F**) Flow cytometry analysis of apoptotic cell rates in POI-GCs after MT and siFOXO3A treatment (n = 4). (**G**) Analysis of ROS production in POI-GCs after MT and siFOXO3A treatment (n = 3). *p < 0.05, **p < 0.01, ***p < 0.001.

The FOXO transcription factor family, which includes FOXO1 and FOXO3, has been shown to modulate the autophagy process [37–39]. In order to elucidate the underlying molecular mechanism of autophagy regulation and cell viability rescue in granulosa cells from individuals with premature ovarian insufficiency (POI-GCs) following melatonin (MT) treatment, we examined the expression of FOXO3A. Surprisingly, we found that FOXO3A was down-regulated in both mRNA and protein levels in the POI group compared to the normal group (Figure 5A, 5B). Additionally, the inactive form of FOXO3A accumulated in the POI group. Upon treating POI-GCs with MT at varying concentrations, we observed a significant increase in FOXO3A protein levels, accompanied by a decrease in phosphorylated FOXO3A (Figure 5C). Moreover, using siRNA to silence FOXO3A significantly reversed the MT-induced increase in autophagic activity (Figure 5D), abolished the protective effects of MT on POI-GC proliferation (Figure 5E), and diminished cell survival (Figure 5F, 5G). Our results indicate that the ability of MT to reverse autophagy insufficiency in POI-GCs is dependent on FOXO3A signaling.

## DISCUSSION

This study has unveiled that granulosa cells (GCs) derived from patients with premature ovarian insufficiency (POI) display a deficiency in autophagy along with reduced levels of melatonin (MT). Notably, our experiments involving MT treatment at varying concentrations revealed that MT elevated autophagy flux and bolstered the viability of POI-GCs. Mechanistically, we observed down-regulated mRNA and protein expression of FOXO3A in POI-GCs. However, treatment with MT resulted in a significant upregulation of FOXO3A and activation of FOXO-dependent transcription. These findings emphasize the pivotal role of MT in regulating autophagy in GCs, especially in the context of POI, and suggest that the MT-FOXO3A signaling pathway could represent a promising therapeutic target for POI treatment.

POI can be classified into two categories: genetic and non-genetic causes. Genetic causes involve mutations in over 50 genes that affect various processes including gonadal development, DNA replication, DNA repair, and hormonal signaling. Non-genetic causes include autoimmune and metabolic disorders, infections, and environmental factors [1, 2, 40]. Therefore, the comprehensive mechanisms underlying POI remain unclear. Recently, autophagy has emerged as an important cellular process for maintaining ovarian function and female reproduction [41, 42]. It has been reported that autophagy regulates the apoptosis of GCs to accelerate follicular atresia [17–20], and insufficient autophagy has been found in GCs from biochemical POI patients [36]. Our study also revealed that autophagy-related genes and proteins are decreased in POI-GCs, which is accompanied by MT deficiency.

It is widely acknowledged that MT efficiently inhibits apoptosis and autophagy in oocytes and GCs under oxidative stress [21–23]. These findings suggest that MT deficiency may be the primary reason for autophagy down-regulation in POI-GCs. Therefore, we conducted an experiment where we treated POI-GCs with exogenous MT in a concentration gradient. Surprisingly, we observed a significant increase in the levels of autophagy-related proteins and an improvement in cell viability. These results, combined with previous data from other studies [27–30], suggest that reversing MT deficiency could potentially serve as a promising clinical treatment for addressing insufficient autophagy in POI.

The mechanisms by which melatonin regulates autophagy have been partially elucidated, involving pathways such as PI3K-Akt-mTOR [31], YAP-Hippo [32], SIRT1 pathway [33]. In our study, we observed a down-regulation of FOXO3A in POI-GCs. FOXO3A is known to encode a master regulator and potent suppressor of ovarian development [2, 43], and its loss of function in mice leads to POI as a result of global follicle activation [44]. Consistent with these findings, we found that MT treatment effectively activated FOXO3A, and silencing FOXO3A significantly attenuated the function of MT. Therefore, the predominant mechanism by which MT regulates autophagy in POI-GCs appears to be through the FOXO3A signaling pathway. However, further research is needed to explore the interplay between MT, FOXO3A, and phosphorylated-FOXO3A, as well as to identify the downstream targets of FOXO3A involved in regulating autophagy. Genomics and proteomics analyses in future studies may contribute to a more comprehensive understanding of the mechanisms underlying POI-GCs. In this study, we successfully demonstrated that the addition of MT supplementation effectively reversed inadequate autophagy, resulting in enhanced viability of POI-GCs by activating the FOXO3A signaling pathway.

## CONCLUSIONS

Our research has revealed that the absence of MT results in the suppression of autophagy in POI-GCs. Furthermore, we have demonstrated that MT treatment efficiently restores autophagy levels and enhances the viability of GCs by activating the FOXO3A signaling pathway. These findings suggest that targeting the MT-FOXO3A axis holds considerable promise as a therapeutic strategy for addressing human POI.

## References

[1] Tucker EJ, Grover SR, Bachelot A, Touraine P, Sinclair AH. Premature Ovarian Insufficiency: New Perspectives on Genetic Cause and Phenotypic Spectrum. Endocr Rev. 2016; 37:609–35. 10.1210/er.2016-1047 27690531

[2] França MM, Mendonca BB. Genetics of ovarian insufficiency and defects of folliculogenesis. Best Pract Res Clin Endocrinol Metab. 2022; 36:101594. 10.1016/j.beem.2021.101594 34794894

[3] Chon SJ, Umair Z, Yoon MS. Premature Ovarian Insufficiency: Past, Present, and Future. Front Cell Dev Biol. 2021; 9:672890. 10.3389/fcell.2021.672890 34041247 PMC8141617

[4] Matsuda F, Inoue N, Manabe N, Ohkura S. Follicular growth and atresia in mammalian ovaries: regulation by survival and death of granulosa cells. J Reprod Dev. 2012; 58:44–50. 10.1262/jrd.2011-012 22450284

[5] Liu YX, Zhang Y, Li YY, Liu XM, Wang XX, Zhang CL, Hao CF, Deng SL. Regulation of follicular development and differentiation by intra-ovarian factors and endocrine hormones. Front Biosci (Landmark Ed). 2019; 24:983–93. 10.2741/4763 30844725

[6] Yang D, Jiang T, Lin P, Chen H, Wang L, Wang N, Zhao F, Wang A, Jin Y. Knock-down of apoptosis inducing factor gene protects endoplasmic reticulum stress-mediated goat granulosa cell apoptosis. Theriogenology. 2017; 88:89–97. 10.1016/j.theriogenology.2016.10.001 27865417

[7] Canipari R, Mangialardo C, Di Paolo V, Alfei F, Ucci S, Russi V, Santaguida MG, Virili C, Segni M, Misiti S, Centanni M, Verga Falzacappa C. Thyroid hormones act as mitogenic and pro survival factors in rat ovarian follicles. J Endocrinol Invest. 2019; 42:271–82. 10.1007/s40618-018-0912-2 29934772

[8] Safdar M, Liang A, Rajput SA, Abbas N, Zubair M, Shaukat A, Rehman AU, Jamil H, Guo Y, Ullah F, Yang L. Orexin-A Regulates Follicular Growth, Proliferation, Cell Cycle and Apoptosis in Mouse Primary Granulosa Cells via the AKT/ERK Signaling Pathway. Molecules. 2021; 26:5635. 10.3390/molecules26185635 34577105 PMC8467508

[9] D’Arcy MS. Cell death: a review of the major forms of apoptosis, necrosis and autophagy. Cell Biol Int. 2019; 43:582–92. 10.1002/cbin.11137 30958602

[10] Pentimalli F. Autophagy in disease: hunger for translation. Cell Death Dis. 2019; 10:247. 10.1038/s41419-019-1419-2 30867407 PMC6416282

[11] Wu C, Zhou L, Yuan H, Wu S. Interconnections among major forms of regulated cell death. Apoptosis. 2020; 25:616–24. 10.1007/s10495-020-01632-2 32889605

[12] Shliapina VL, Yurtaeva SV, Rubtsova MP, Dontsova OA. At the Crossroads: Mechanisms of Apoptosis and Autophagy in Cell Life and Death. Acta Naturae. 2021; 13:106–15. 10.32607/actanaturae.11208 34377561 PMC8327148

[13] Kist M, Vucic D. Cell death pathways: intricate connections and disease implications. EMBO J. 2021; 40:e106700. 10.15252/embj.2020106700 33439509 PMC7917554

[14] Shen M, Jiang Y, Guan Z, Cao Y, Li L, Liu H, Sun SC. Protective mechanism of FSH against oxidative damage in mouse ovarian granulosa cells by repressing autophagy. Autophagy. 2017; 13:1364–85. 10.1080/15548627.2017.1327941 28598230 PMC5584866

[15] Yadav PK, Tiwari M, Gupta A, Sharma A, Prasad S, Pandey AN, Chaube SK. Germ cell depletion from mammalian ovary: possible involvement of apoptosis and autophagy. J Biomed Sci. 2018; 25:36. 10.1186/s12929-018-0438-0 29681242 PMC5911955

[16] Yadav AK, Yadav PK, Chaudhary GR, Tiwari M, Gupta A, Sharma A, Pandey AN, Pandey AK, Chaube SK. Autophagy in hypoxic ovary. Cell Mol Life Sci. 2019; 76:3311–22. 10.1007/s00018-019-03122-4 31062072 PMC11105528

[17] Choi J, Jo M, Lee E, Choi D. Induction of apoptotic cell death via accumulation of autophagosomes in rat granulosa cells. Fertil Steril. 2011; 95:1482–6. 10.1016/j.fertnstert.2010.06.006 20630503

[18] Zheng Y, Ma L, Liu N, Tang X, Guo S, Zhang B, Jiang Z. Autophagy and Apoptosis of Porcine Ovarian Granulosa Cells During Follicular Development. Animals (Basel). 2019; 9:1111. 10.3390/ani9121111 31835576 PMC6940823

[19] Zhang JQ, Ren QL, Chen JF, Gao BW, Wang XW, Zhang ZJ, Wang J, Xu ZJ, Xing BS. Autophagy Contributes to Oxidative Stress-Induced Apoptosis in Porcine Granulosa Cells. Reprod Sci. 2021; 28:2147–60. 10.1007/s43032-020-00340-1 33079330

[20] Tang Z, Xu R, Zhang Z, Shi C, Zhang Y, Yang H, Lin Q, Liu Y, Lin F, Geng B, Wang Z. HIF-1α Protects Granulosa Cells From Hypoxia-Induced Apoptosis During Follicular Development by Inducing Autophagy. Front Cell Dev Biol. 2021; 9:631016. 10.3389/fcell.2021.631016 33553188 PMC7862574

[21] Shen M, Cao Y, Jiang Y, Wei Y, Liu H. Melatonin protects mouse granulosa cells against oxidative damage by inhibiting FOXO1-mediated autophagy: Implication of an antioxidation-independent mechanism. Redox Biol. 2018; 18:138–57. 10.1016/j.redox.2018.07.004 30014903 PMC6068202

[22] Chen Z, Lei L, Wen D, Yang L. Melatonin attenuates palmitic acid-induced mouse granulosa cells apoptosis via endoplasmic reticulum stress. J Ovarian Res. 2019; 12:43. 10.1186/s13048-019-0519-z 31077207 PMC6511168

[23] Tao JL, Zhang X, Zhou JQ, Li CY, Yang MH, Liu ZJ, Zhang LL, Deng SL, Zhang L, Shen M, Liu GS, Liu HL. Melatonin Alleviates Hypoxia-Induced Apoptosis of Granulosa Cells by Reducing ROS and Activating MTNR1B-PKA-Caspase8/9 Pathway. Antioxidants (Basel). 2021; 10:184. 10.3390/antiox10020184 33525391 PMC7911142

[24] Tamura H, Kawamoto M, Sato S, Tamura I, Maekawa R, Taketani T, Aasada H, Takaki E, Nakai A, Reiter RJ, Sugino N. Long-term melatonin treatment delays ovarian aging. J Pineal Res. 2017; 62. 10.1111/jpi.12381 27889913

[25] Zhang L, Zhang Z, Wang J, Lv D, Zhu T, Wang F, Tian X, Yao Y, Ji P, Liu G. Melatonin regulates the activities of ovary and delays the fertility decline in female animals via MT1/AMPK pathway. J Pineal Res. 2019; 66:e12550. 10.1111/jpi.12550 30597622

[26] Reiter RJ, Sharma R, Romero A, Manucha W, Tan DX, Zuccari DA, Chuffa LGA. Aging-Related Ovarian Failure and Infertility: Melatonin to the Rescue. Antioxidants (Basel). 2023; 12:695. 10.3390/antiox12030695 36978942 PMC10045124

[27] Kandemir YB, Konuk E, Behram M, Sindel M. Effect of Melatonin on the Expression of VEGF-A and on the Degeneration of Follicle Reserve in Rat Ovary. Eurasian J Med. 2018; 50:160–3. 10.5152/eurasianjmed.2018.17361 30515035 PMC6263225

[28] Yang Q, Zhu J, Luo X, Li F, Cong L, Wang Y, Sun Y. Melatonin attenuates cadmium-induced ovulatory dysfunction by suppressing endoplasmic reticulum stress and cell apoptosis. Reprod Biol Endocrinol. 2019; 17:61. 10.1186/s12958-019-0502-y 31358006 PMC6661738

[29] Qi MK, Sun TC, Yang LY, He JL, Guo YM, Wang HB, Wang HP. Therapeutic Effect of Melatonin in Premature Ovarian Insufficiency: Hippo Pathway Is Involved. Oxid Med Cell Longev. 2022; 2022:3425877. 10.1155/2022/3425877 36017238 PMC9398856

[30] Feng J, Ma WW, Li HX, Pei XY, Deng SL, Jia H, Ma WZ. Melatonin prevents cyclophosphamide-induced primordial follicle loss by inhibiting ovarian granulosa cell apoptosis and maintaining AMH expression. Front Endocrinol (Lausanne). 2022; 13:895095. 10.3389/fendo.2022.895095 35992124 PMC9381702

[31] Wu D, Zhao W, Xu C, Zhou X, Leng X, Li Y. Melatonin suppresses serum starvation-induced autophagy of ovarian granulosa cells in premature ovarian insufficiency. BMC Womens Health. 2022; 22:474. 10.1186/s12905-022-02056-7 36434569 PMC9700896

[32] Xu H, Bao X, Kong H, Yang J, Li Y, Sun Z. Melatonin Protects Against Cyclophosphamide-induced Premature Ovarian Failure in Rats. Hum Exp Toxicol. 2022; 41:9603271221127430. 10.1177/09603271221127430 36154502

[33] Ma M, Chen XY, Li B, Li XT. Melatonin protects premature ovarian insufficiency induced by tripterygium glycosides: role of SIRT1. Am J Transl Res. 2017; 9:1580–602. 28469767 PMC5411910

[34] Ke H, Tang S, Guo T, Hou D, Jiao X, Li S, Luo W, Xu B, Zhao S, Li G, Zhang X, Xu S, Wang L, et al. Landscape of pathogenic mutations in premature ovarian insufficiency. Nat Med. 2023; 29:483–92. 10.1038/s41591-022-02194-3 36732629 PMC9941050

[35] Klionsky DJ, Abdel-Aziz AK, Abdelfatah S, Abdellatif M, Abdoli A, Abel S, Abeliovich H, Abildgaard MH, Abudu YP, Acevedo-Arozena A, Adamopoulos IE, Adeli K, Adolph TE, et al. Guidelines for the use and interpretation of assays for monitoring autophagy (4th edition). Autophagy. 2021; 17:1–382. 10.1080/15548627.2020.1797280 33634751 PMC7996087

[36] Shao T, Ke H, Liu R, Xu L, Han S, Zhang X, Dang Y, Jiao X, Li W, Chen ZJ, Qin Y, Zhao S. Autophagy regulates differentiation of ovarian granulosa cells through degradation of WT1. Autophagy. 2022; 18:1864–78. 10.1080/15548627.2021.2005415 35025698 PMC9450966

[37] Zhou J, Liao W, Yang J, Ma K, Li X, Wang Y, Wang D, Wang L, Zhang Y, Yin Y, Zhao Y, Zhu WG. FOXO3 induces FOXO1-dependent autophagy by activating the AKT1 signaling pathway. Autophagy. 2012; 8:1712–23. 10.4161/auto.21830 22931788 PMC3541283

[38] Warr MR, Binnewies M, Flach J, Reynaud D, Garg T, Malhotra R, Debnath J, Passegué E. FOXO3A directs a protective autophagy program in haematopoietic stem cells. Nature. 2013; 494:323–7. 10.1038/nature11895 23389440 PMC3579002

[39] Lee JW, Nam H, Kim LE, Jeon Y, Min H, Ha S, Lee Y, Kim SY, Lee SJ, Kim EK, Yu SW. TLR4 (toll-like receptor 4) activation suppresses autophagy through inhibition of FOXO3 and impairs phagocytic capacity of microglia. Autophagy. 2019; 15:753–70. 10.1080/15548627.2018.1556946 30523761 PMC6526818

[40] Qin Y, Jiao X, Simpson JL, Chen ZJ. Genetics of primary ovarian insufficiency: new developments and opportunities. Hum Reprod Update. 2015; 21:787–808. 10.1093/humupd/dmv036 26243799 PMC4594617

[41] Peters AE, Mihalas BP, Bromfield EG, Roman SD, Nixon B, Sutherland JM. Autophagy in Female Fertility: A Role in Oxidative Stress and Aging. Antioxid Redox Signal. 2020; 32:550–68. 10.1089/ars.2019.7986 31892284

[42] Gao H, Khawar MB, Li W. Essential role of autophagy in resource allocation during sexual reproduction. Autophagy. 2020; 16:18–27. 10.1080/15548627.2019.1628543 31203720 PMC6984600

[43] Barberino RS, Macedo TJS, Lins TL, Menezes VG, Silva RLS, Monte APO, Palheta RC Jr, Smitz JEJ, Matos MHT. Immunolocalization of melatonin receptor type 1 in the sheep ovary and involvement of the PI3K/Akt/FOXO3a signaling pathway in the effects of melatonin on survival and *in vitro* activation of primordial follicles. Mol Reprod Dev. 2022; 89:485–97. 10.1002/mrd.23639 35943024

[44] Liu L, Rajareddy S, Reddy P, Du C, Jagarlamudi K, Shen Y, Gunnarsson D, Selstam G, Boman K, Liu K. Infertility caused by retardation of follicular development in mice with oocyte-specific expression of Foxo3a. Development. 2007; 134:199–209. 10.1242/dev.02667 17164425

